# Barriers and facilitators to initiating and adhering to harm reduction services among people who inject drugs in the United States: a systematic review

**DOI:** 10.1186/s12954-025-01376-9

**Published:** 2026-02-12

**Authors:** Carrie L. Nacht, Britt Skaathun, Kristen Ogarrio, Reanna Durbin-Matrone, Laura Wright, Rick Reich, Kat Reich, Kristefer Stojanovski

**Affiliations:** 1 School of Public Health, San Diego State University, San Diego, CA USA; 2 Division of Infections Disease and Global Public Health La Jolla, Herbert Wertheim School of Public Health and Human Longevity Science, University of California San Diego, 9500, Gilman Drive, La Jolla, 92093 USA; 3 Department of Epidemiology, Celia Scott Weatherhead School of Public Health and Tropical Medicine, Tulane University, New Orleans, LA USA; 4 Celia Scott Weatherhead School of Public Health and Tropical Medicine, Tulane University, New Orleans, LA USA; 5 Rudolph Matas Library of Health Sciences, Tulane University, New Orleans, LA USA; 6 Trac B Exchange, Las Vegas, NV USA; 7 Department of Social, Behavioral, and Population Sciences, Celia Scott Weatherhead School of Public Health and Tropical Medicine, Tulane University, New Orleans, LA USA

**Keywords:** People who inject drugs, Substance abuse, Harm reduction, Substance use treatment services

## Abstract

**Background:**

Harm reduction services are inequitably accessible and efficient among people with a history of injection drug use (PWHID), particularly among those belonging to vulnerable communities. This systematic review identifies barriers and facilitators to accessing and adhering to harm reduction services among PWHID in priority populations to gain a deeper understanding of the intersectional barriers to these services.

**Methods:**

A systematic search was conducted using four databases (PubMed/MEDLINE, Scopus, CINAHL, and Web of Science) to locate articles examining barriers and/or facilitators associated with substance use harm reduction services among marginalized populations (e.g., racial/ethnic minorities, sexual/gender minorities, people living with HIV, veterans, etc.) in the United States. Study characteristics and key findings were extracted from studies and thematically analyzed.

**Results:**

This review identified 25 articles that examined factors associated with initiation and adherence to harm reduction services for PWHID. The harm reduction services most identified were medications for opioid use disorders (e.g., naloxone, buprenorphine) and syringe service programs. Factors were individual-level factors (e.g., motivation, stigma, previous negative experiences), as well as service-level barriers (e.g., accessibility, distance, setting). Many studies identified participant sociodemographic characteristics (e.g., race, age, sexuality) as a facilitator or barrier, although these factors are proxies for systemic and/or structural barriers and should not necessarily be considered as intervention targets.

**Conclusion:**

Incorporating harm reduction services as part of integrated, wraparound services in other healthcare settings may reduce barriers such as convenience, inaccessibility due to geography or transportation, stigma, and encourage service utilization as a result.

**Supplementary Information:**

The online version contains supplementary material available at 10.1186/s12954-025-01376-9.

## Introduction

Substance use disorders in the United States have reached a critical juncture, driven by the convergence of multiple public health crises. According to the Centers for Disease Control and Prevention (CDC) reports, the opioid epidemic continues to ravage communities, with over 107,000 drug overdose deaths reported in 2023, a figure primarily driven by synthetic opioids like fentanyl [1]. There are several highly effective harm reduction strategies, which aim to reduce the risk of severe health consequences without requiring individuals to be completely or permanently abstinent [2]. Some harm reduction strategies, such as syringe exchange programs and naloxone distribution, have been shown to significantly reduce the risks associated with substance use, including the transmission of infectious diseases and fatal overdoses [3, 4]. The implementation of naloxone programs has been associated with a marked decrease in opioid overdose deaths, with one study demonstrating a 14% reduction in overdose mortality rates in communities with active naloxone distribution [5–7].

Despite their proven efficacy, harm reduction services are not equitably accessible or efficient across marginalized populations (e.g., racial/ethnic minorities, sexual/gender minorities, those with unstable housing, people living with HIV, etc.). For example, previous studies have shown that these racial/ethnic minority communities have less engagement in harm reduction services compared to White populations [8–11]. This racial disparity is often attributable to numerous factors, including stigma, systemic racism, socioeconomic disparities, and mistrust of healthcare systems [12–19]. As such, while the age-adjusted rate of overdose deaths has decreased among White adults, overdose deaths have increased among nearly every racial/ethnic minority group from 2022 to 2023 [20]. Likewise, members of the lesbian, gay, bisexual, transgender, and queer (LGBTQ) community are over twice as likely to experience a substance use disorder compared to heterosexual adults, and transgender individuals are almost four times as likely compared to their cisgender counterparts [21, 22]. LGBTQ individuals face many barriers to substance use treatment, including services not addressing lived experiences (e.g., heteronormative beliefs, misgendering) [23], which can be perceived as discriminatory or exclusionary, and may reduce treatment adherence [24].

Individuals who exist at the nexus of several minoritized identities (e.g., unhoused, sexual/gender minority, racial/ethnic minority, people living with HIV) must be considered using an intersectional lens. The intersectional paradigm attempts to highlight how different identities (e.g., race, gender, socioeconomic status) cannot be considered individually, but instead examines the intersection of these identities to provide an accurate estimate of privilege or oppression [25–27]. Those belonging to multiple marginalized or minoritized groups are likely encountering *intersectional stigma* related to said identities, which negatively impacts health through structural and interpersonal systems of oppression [28–31]. As such, while factors such as race, sexuality, age, and ethnicity may be cited as barriers to accessing harm reduction services, they should be considered indicators of broader structural inequities. These inequities are shaped by inadequate systems (e.g., capitalism, healthcare infrastructure) and enduring legacies of racism and misogyny to create and sustain disparities in attainable and effective care.

The inequitable availability of impactful harm reduction services for vulnerable communities highlights the urgent need for comprehensive, evidence-based approaches tailored to these populations to address the growing substance use epidemic in the U.S. While summaries of barriers and facilitators to harm reduction services for substance use disorder (SUD) exist, or have been noted among specific populations, an overarching review summarizing barriers and facilitators for people with a history of injection drug use (PWHID) in the U.S. is needed. This is a critical first step before implementing effective harm reduction services. We conducted a systematic review to identify specific barriers and facilitators related to both initiation and adherence of harm reduction services experienced by PWHID in general and PWHID with intersectional identities. In this review, we synthesized our findings by identifying targets upon which future researchers and clinicians may intervene to increase the use of harm reduction services. Identifying these intervention targets will allow for purposeful actions to expand harm reduction services to vulnerable populations to ensure life-saving interventions reach all disproportionately impacted populations.

## Methods

Using the Preferred Reporting Items for Systematic Reviews and Meta-Analyses (PRISMA) criteria [32], we conducted a systematic review of the scientific literature that examined the barriers and facilitators associated with accessing and utilizing substance abuse harm reduction services among disproportionately impacted populations. The search was conducted on June 3, 2024, and June 6, 2024, using four databases: PubMed/MEDLINE, Scopus, CINAHL, and Web of Science. Search terms were determined by using keywords associated with harm reduction services, including “substance use,” “needle exchange,” “opioids,” and “drug screening,” and terms related explicitly to populations disproportionately impacted by substance use, such as “LGBTQ + populations,” “minority groups,” and "vulnerable populations”. The full Medical Subject Headings (MeSH) search terms and results are found in Supplementary Table 1.

### Inclusion and exclusion criteria

The inclusion criteria were as follows: (1) examined harm reduction services, (2) examined barriers and/or facilitators to harm reduction services, (3) based in the U.S., (4) human subjects as participants, (5) written in English or Spanish (given the team’s language skills), and (6) peer-reviewed observational study design. Exclusion criteria included: (1) did not include PWHID, (2) did not include any discussion or analysis of vulnerable populations (e.g., LGBTQ + population, experiencing homelessness, racial/ethnic minorities, sex workers, incarcerated, etc.), and (3) the study did not investigate barriers or facilitators to harm reduction services as an outcome.

### Screening and review

**Fig. 1:**
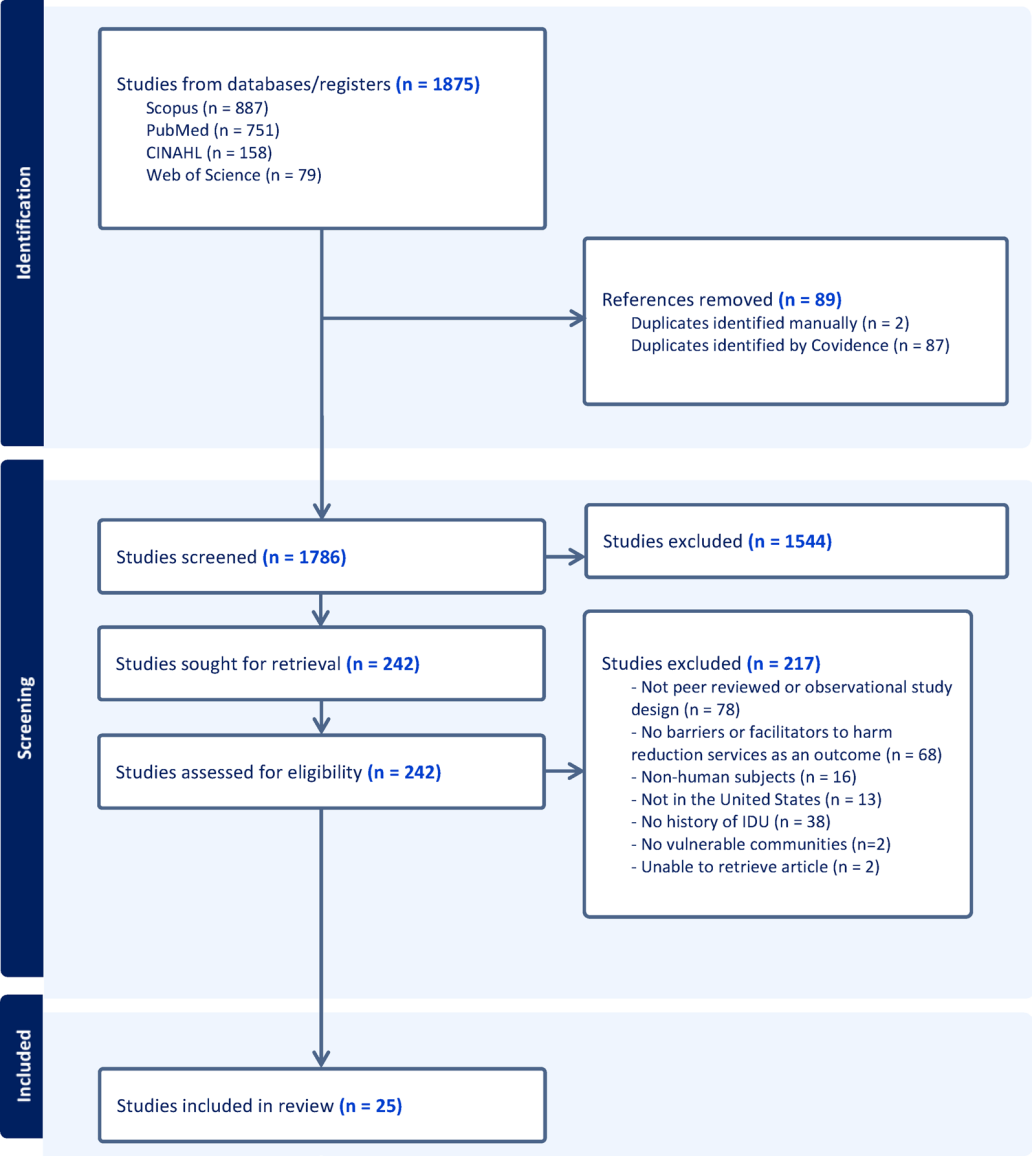
PRISMA diagram

The search was conducted on June 3, 2024, using PubMed/MEDLINE and CINAHL, and on June 6, 2024, on Scopus and Web of Science. After conducting the search, there were two rounds of review: title and abstract screening, which did not require citing reasons for exclusion, and full-text review, in which reasons for exclusion must be identified [32]. This systematic search yielded 1875 articles, which were imported into Covidence [33], an online workflow platform that has systematized review processes. After removing 89 duplicates, 1786 articles progressed to title and abstract screening. Three independent reviewers (RDM, KO, and CN) screened the titles and abstracts in duplicate. We removed 1544 studies that did not meet the inclusion criteria, leaving 242 for full-text review. During full-text review (RDM, KO, and CN), 217 studies were excluded for the following reasons: articles were not peer reviewed or were experimental study designs (n = 78), did not assess barriers or facilitators to harm reduction services as an outcome (n = 68), no PWHID (n = 38), non-human subjects (n = 16), not in the U.S. (n = 13), no discussion or analysis of vulnerable communities (n = 2), and two articles were unable to be retrieved. All conflicts were resolved via discussions between reviewers to come to a consensus on whether to include or exclude an article identified in the search. A total of 25 studies were included in the final analysis. Figure 1 displays the PRISMA flowchart of the screening process.

### Data extraction

Data were extracted from the final sample of articles included using Elicit software [34], an artificial intelligence tool trained on a dataset of scientific research articles, as a way to expedite the data extraction. Several studies have examined Elicit in the context of literature reviews and have found it to be a valuable, complementary, and time-saving tool for systematic reviews, but it does require checking for accuracy [35–37], which our team did in both an extraction validation step and a reviewer checking step. This technique has been used in another literature review [38].

Each article’s PDF was uploaded into Elicit to conduct data extraction. The prompts in Elicit are predetermined a priori (e.g., study design, author(s), publication year, study population, data sources, harm reduction services, etc.). Additionally, we developed prompts a posteriori for our specific research questions to extract information on the barriers and facilitators to substance use harm reduction services. The prompts read as follows: “Extract information from the results related to barriers/facilitators to harm reduction services.” After the data is extracted, Elicit identifies the area from which the information is extracted. All extracted data by Elicit were reviewed for accuracy by one of three reviewers (CN, KO, or RDM) to ensure accurate extraction, consistent reporting, and appropriate scientific writing (e.g., outcomes and independent variables were extracted from the methods, and statistics of results were extracted from the results).

Approval by the university's Institutional Review Boards (IRB) was not required.

### Data synthesis

To synthesize data, the included studies were categorized depending on whether they investigated *initiation* to harm reduction services or *adherence* to harm reduction services. Articles were also categorized by study design, harm reduction service, geographic location, and sub-population. After data extraction validation, factors associated with initiation and adherence were reviewed and quantified. Factors were then systematically organized into being either positively or negatively associated with the respective harm reduction service (either initiation or adherence) specified in the study.

### Quality assessment

Two reviewers (CN, KO) independently assessed the studies that were included in the final analysis to assess risk of bias. Cross-sectional studies were assessed using the Joanna Briggs Institute (JBI) Critical Appraisal Checklist for Analytical Cross Sectional Studies [39], an 8-item checklist including items such as “*Was the exposure measured in a valid and reliable way?*” and “*Were strategies to deal with confounding factors stated?*”. Cohort studies were assessed using the 11-item JBI Critical Appraisal Checklist for Cohort Studies [40]. Sample items include “*Were the two groups similar and recruited from the same population?*” and “*Was the follow up time reported and sufficient to be long enough for outcomes to occur?*”. Qualitative studies were assessed for bias using the Critical Appraisal Skills Programme (CASP) Qualitative Studies Checklist [41]. This checklist was comprised of 11 items, including “*Is a qualitative methodology appropriate?*” and “Has the relationship between researcher and participants been adequately considered?”.

Reviewers responded to each question in the three scales with “Yes” (a value of 1), “No” (a value of 0), or not applicable (NA). Each study was given a final score from the proportion of responses with “Yes” divided by the total number of questions that did not have “NA” as an answer. A score greater than or equal to 85% was deemed “Good”, between 70 and 85% was “Fair”, and less than 70% was “Poor”. After independently assessing the articles for risk of bias, the two reviewers met to discuss any discrepancies until agreeing upon the study quality.

## Results

### Description of studies

Table 1 presents the study characteristics for the 25 studies included in this analysis [11, 42–65]. Eighteen of the studies employed a cross-sectional design [42, 44, 46–48, 50, 52–56, 58–63, 65], six were qualitative [43, 45, 49, 51, 57, 64], and one was a cohort design [11]. Four studies were conducted on a national scale [11, 50, 56, 60], and the remaining studies were conducted in various U.S. states, such as three studies conducted only in California [46, 54, 58], three studies only in Maryland [49, 51, 52], and three studies only in New York state [44, 53, 59]. When assessing the quality of studies, the majority were deemed “Good” (52%), six were deemed “Fair” (24%), and six were deemed “Poor” (24%). Studies were not excluded from this analysis based on quality.

**Table 1:** Study characteristics and quality assessment (n = 25)

First author	Design	Location	Sample size	Specific population(s)	Health service(s)	Quality assessment
Anastario [42]	Cross-Sectional	MT	51	American Indian patients	1. Sterile syringes	Poor
Barnett [11]	Cohort	Nationwide	23,370	Economically disadvantaged, disabled Medicare beneficiaries	1. Hospitalization 2. Outpatient care 3. Emergency room care 4. Buprenorphine 5. Naloxone 6. Benzodiazepine	Fair
Bass [43]	Qualitative	PA	20	Economically disadvantaged and homeless adults	1. Drug test strips	Good
Beidelman [44]	Cross-Sectional	NY	475	–	1. Supporting services 2. Maintenance 3. Primary care	Good
Bonar [45]	Qualitative	OH, MI	90	–	1. Test shots 2. Skin cleaning before injection	Poor
Brecht [46]	Cross-sectional	CA	618	–	1. Methadone	Fair
Chueng [47]	Cross-Sectional	FL	1,272	–	1. SSP 2. Naloxone	Fair
Costenbader [48]	Cross-Sectional	NC	851	–	1. Syringe distribution	Good
Gryczynski (2013) [49]	Qualitative	MD	80	–	1. Buprenorphine 2. Methadone	Fair
Gryczynski (2011) [50]	Cross-Sectional	Nationwide	28,920	–	1. Methadone	Good
Heidari [51]	Qualitative	MD	24	Patients with multimorbid chronic and mental health conditions	1. Outpatient treatment 2. MOUD 3. ER visits	Good
Jones [52]	Cross-Sectional	MD	499	Impoverished, homeless, African Americans, and those involved in sex work	1. Naloxone	Poor
Kang [53]	Cross-Sectional	NY	220	HIV-infected African American and Hispanic patients	1. Methadone 2. Outpatient programs 3. Residential treatment 4. ED/ER visits	Good
Kim [54]	Cross-Sectional	CA	1,670	–	1. Sterile syringes	Poor
Krawczyk (2022) [55]	Cross-Sectional	DC, ME, MD, MI, NJ, NM, NY, PA, TN, WV	511	–	1. MOUD only 2. MOUD&SSP 3. SSP only	Poor
Krawczyk (2017) [56]	Cross-Sectional	Nationwide	94,202	–	1. Buprenorphine 2. Methadone	Good
Leston [57]	Qualitative	Nationwide	32	American Indians / Alaskan Native adults	1. Multidisciplinary healthcare team 2. SSP 3. Medication assisted treatment	Good
Martinez [58]	Cross-Sectional	CA	1,430	Mexican American patients	1. Methadone 2. Outpatient treatment 3. Residential treatment 4. Self-help	Good
Nolen [59]	Cross-Sectional	NY	79,555	–	1. Naloxone	Good
Pro [60]	Cross-Sectional	Nationwide	20,121	Patients experiencing homelessness	1. Outpatient treatment	Good
Reynolds [61]	Cross-Sectional	AK	645	–	1. Primary care 2. ED/ER visit	Fair
Salow [62]	Cross-Sectional	WA	899	–	1. SSP	Fair
Shrestha [63]	Cross-Sectional	MA	429	–	1. Methadone 2. Buprenorphine 3. Naloxone	Poor
VanderWaal [64]	Qualitative	Midwestern cities	19	–	1. Methadone 2. Syringe distribution 3. Needle cleaning supplies 4. National prevention campaigns 5. Mass-media prevention campaigns	Good
Williams [65]	Cross-Sectional	PA	2,599	–	1. Syringe distribution	Good

A wide variety of harm reduction services were assessed in the included studies. The most common harm reduction services measured were medications for opioid use disorders (MOUDs) (n = 13), such as methadone (n = 8), naloxone/naltrexone (n = 4), buprenorphine (n = 3). Other harm reduction services included syringe services (e.g., distribution, exchanges, supplies) (n = 11), outpatient treatment (n = 4), residential treatment (n = 2), emergency room (ER) visit or hospitalization (n = 4), primary care (n = 2), and mass-media prevention campaigns (n = 2). One study each assessed any drug treatment in the past 6 months (including 12-step self-help program, methadone detoxification and maintenance, outpatient treatment) [58], other service utilization (e.g., social services, case management and linkage to outside services, HIV/STI care, mental health services, basic needs) [44], drug testing strips [43], and skin cleaning before injection (Table 1) [45].

Twenty-one studies only examined factors related to *initiation* of harm reduction services, such as receiving a prescription for MOUDs, sterile syringes, or starting treatment (e.g., primary care, ER, inpatient, or outpatient) [42–50, 52–59, 61–65]. On the other hand, three studies examined factors influencing treatment adherence, such as treatment outcomes, engagement, and satisfaction with care [11, 51, 60]. One study investigated factors associated with both initiation and adherence to service use [46].

### Participant characteristics related to harm reduction service usage

Nineteen of the twenty-five studies (76%) included in this analysis revealed disparities in harm reduction services across non-intervenable patient characteristics (e.g., race, age, gender). These factors, along with their respective effect sizes, are listed in Table 2. It should be noted that these participant characteristics should be considered as contextual factors arising from the structural and systemic environment in the U.S., rather than as intervention opportunities. Furthermore, many of these factors had complex associations with harm service initiation and adherence and were not explicitly identified as either protective or risk factors.

**Table 2:** Demographic and socioeconomic disparities associated with harm reduction service initiation and adherence (n = 25)

First author (Year)	services	Outcome	Factors associated with outcome*	Findings
Anastario (2017)	1. Sterile syringes	1. Obtaining new syringes	–	–
Barnett (2023)	1. Hospitalization 2. Outpatient care 3. Emergency room care 4. Buprenorphine 5. Naloxone 6. Benzodiazepine	1. Health care utilization 180 days after index events	1. Race/ethnicity	1. Buprenorphine was prescribed less to Black patients than White patients (aD = −8.7, 95% CI −11.3, −6.0) 2. Buprenorphine was prescribed less to Hispanic patients than White patients (aD = −4.2, 95% CI −6.7, −1.8) 3. Black patients that did receive a buprenorphine prescription received a lower days’ supply than White patients (aD = −23.4 days, 95% CI −32.5, 14.2) 4. A smaller proportion of Black recipients of buprenorphine compared to White recipients were retained in treatment at least 150 days (30.1% vs. 44.8%, aD = −14.0, 95% CI −20.3, −7.8) 5. Naloxone was prescribed less to Black patients than White patients (aD = −6.7, 95% CI −9.5, −3.7) 6. Naloxone was prescribed more to Hispanic patients than Black patients (aD = 4.3, 95% CI 1.5, 7.1) 7. Benzodiazepine was prescribed less to Black patients than White patients (aD = −14.1, 95% CI –16.7, −11.6) . Benzodiazepine was prescribed less to Hispanic patients than White patients (aD = −6.7, 95% CI −8.4, −5.1) 8. Benzodiazepine was prescribed more to Hispanic patients than Black patients (aD = 7.4, 95% CI 4.5, 10.3) 9. Less index events were followed by ambulatory visits among Black patients compared to White patients (average number of visits 6.6 and 7.6 respectively, aD = −0.9, 95% CI −1.3, −0.6) 10. Less index events were followed by ambulatory visits among Hispanic patients compared to White patients (average number of visits 6.7 and 7.6 respectively, aD = −0.6, 95% CI −0.9, −0.3) 11. After the index event, more Black patients had emergency room visits (3.2 ± 6.8 visits vs. 2.4 ± 4.1) and hospitalizations (1.2 ± 1.9 vs. 0.9 ± 1.5) compared to White patients 12. In 2020–2021, Black patients received either buprenorphine or methadone less often than White patients (aD = −5.1, 95% CI −8.5, −1.8) 13. In 2020–2021, Hispanic patients received either buprenorphine or methadone less often than White patients (aD = −4.8, 95% CI −8.2, −1.4)
Bass (2022)	1. Drug test strips	1. Using fentanyl drug testing strips before IDU	–	–
Beidelman (2023)	1. Supporting services 2. Maintenance 3. Primary care	1. Odds of using ancillary services - Cluster 1: no/low use - Cluster 2: high use of support, maintenance, and primary care - Cluster 3: high use of all	1. Insurance ( +) 2. Race/ethnicity	1. PWHID who identified as being White had lower odds (aOR = 0.19, 95% CI 0.07, 0.50) of being in Cluster 3 (high use of all services) than Cluster 1 (no/low use of services) 2. Medicaid recipients had higher odds (aOR = 2.89, 95% CI 1.01, 8.36) of being in Cluster 3 (high use of all services) than Cluster 1 (no/low use of services)
Bonar (2014)	1. Test shots 2. Skin cleaning before injection	1. Using test shots prior to IDU 2. Skin cleaning prior to IDU	–	–
Brecht (1993)	1. Methadone	1. Reason for entering methadone maintenance (MM) treatment 2. Variables associated with treatment effectiveness	1. Race/ethnicity 2. Gender	Reason for Entering 1. A larger proportion of Chicano male participants reported entering MM due to legal coercion compared to Anglo males (53% vs. 41% for), while the opposite was true for females (24% for Anglo females, 14% Chicana participants) 2. More nonlegal reasons (e.g., tired of heroin lifestyle, family/friends, lack of funds, health considerations) for entering MM were reported by Anglo males than Chicano males (84% vs. 81%), while the opposite was true for females (90% Anglo females, 98% Chicana females) 3. A small percentage of participants reported entering MM for legal reasons only (16% Anglo males, 19% Chicano male; 10% Anglo females, 2% Chicana females) Treatment Outcomes 1. Percent of time dealing for profit and using alcohol heavily both significantly decreased over time and differed significantly by sex (*p* < 0.01) *2. See *Table 3* for results on time/ethnicity/legal coercion interaction results*
Chueng (2022)	1. SSP 2. Naloxone	1. Odds of SSP visits and naloxone utilization by risk class: - LIHS: Low Injection, High Sexual risk - HIMS: High Injection, Moderate Sexual risk - LILS: Low Injection, Low Sexual risk	1. LGBT orientation 2. Housing 3. Gender	1. Participants in HIMS had higher SSP visits (21.1 vs. 11.8, *p* = 0.001) and naloxone accessed (15.7 per 100 person years vs. 6.2 per 100 person years, *p* = 0.002) compared to LILS members 2. Participants who reported LGBT orientation had higher odds of being in LIHS than LILS (aOR = 5.85, 95% CI 3.01, 11.34) 3. Participants who reported LGBT orientation had higher odds of being in HIMS than LILS (aOR = 2.21, 95% CI 1.34, 3.64) 4. Those who reported LGBT orientation had higher odds of being in LIHS than HIMS (aOR = 2.65, 95% CI 1.25, 5.59) 5. Participants who reported unstable housing had higher odds of being in HIMS than LILS (aOR = 2.04, 95% CI 1.44, 2.89) 6. Those who reported unstable housing had lower odds of being in LIHS than HIMS (aOR = 0.44, 95% CI 0.19, 0.99) 7. Men were had lower odds of being in HIMS than LILS (aOR = 0.66, 95% CI 0.46, 0.96)
Costenbader (2010)	1. Syringe distribution	1. Odds of pharmacy as main source of syringe	1. Recruitment location 2. Race/ethnicity	1. Being recruited in Raleigh was associated with higher odds (aOR 1.70, 95% CI 1.17–2.48) of having a pharmacy be the primary source of syringe purchases 2. Being African American was associated with lower odds (aOR 0.22, 95% 0.15–0.34) of having a pharmacy be the primary source of syringe purchases
Gryczynski (2011)	1. Methadone	1. Odds of delay in admission to outpatient methadone treatment	1. Race/ethnicity 2. Education 3. Insurance	1. Black participants had higher odds of experiencing admission delays vs non-Hispanic White participants (OR = 1.358, 95% CI 1.241, 1.486) 2. Hispanic participants had higher odds of experiencing delays vs non-Hispanic White participants (OR = 1.170, 95% CI 1.055,1.298) 3. Participants with less than a high school education had higher odds of experiencing admission delays relative to their high school-educated counterparts (OR = 1.106, 95% CI 1.033, 1.185) 4. There were higher odds of admission delays for paying for services, including insurance coverage (OR = 2.023, 95% CI 1.681–2.434, *p* < 0.001), noninsurance government payments (OR = 1.752, 95% CI 1.470–2.089, *p* < 0.001), and treatment provided free of charge (OR = 3.698, 95% CI 2.497–5.476, *p* < 0.001) relative to self-payment
Gryczynski (2013)	1. Buprenorphine 2. Methadone	1. Reasons for using buprenorphine instead of methadone 2. Reasons for not using methadone	–	–
Heidari (2024)	1. Outpatient treatment 2. MOUD 3. ER visits	Engagement and satisfaction with services	–	–
Jones (2023)	1. Naloxone	Odds of always/often carrying naloxone	1. Age 2. Gender 3. Education 4. Housing 5. Race/ethnicity	1. Participants who were older had higher odds of carrying naloxone compared to younger participants (aOR = 1.03, 95% CI 1.00, 1.06) 2. There were higher odds of females carrying naloxone than males (aOR = 1.69, 95% CI 1.03, 2.77) 3. Participants who had some college education were over two times as likely to carry naloxone compared to those with less than a high school education (aOR = 2.06, 95% CI 1.09, 3.91) 4. People who were experiencing homelessness were more likely to carry naloxone compared to those who had stable housing (aOR = 1.94, 95% CI 1.16, 3.24) 5. There were lower odds for Black participants to carry naloxone compared to White participants (aOR = 0.39, 95% CI 0.22, 0.69)
Kang (2006)	1. Methadone 2. Outpatient programs 3. Residential treatment 4. ER visits	1. Odds of any drug treatment enrollment 2. Comparison of enrollment in drug treatment and ER visits between African American and Hispanic patients	1. Race/ethnicity	1. Compared to African American participants, Hispanic participants were more likely to utilize drug treatment programs (*p* < 0.001) and visit the emergency room (*p* < 0.05)
Kim (2015)	1. Sterile syringes	1. Likelihood of using new sterile needles	–	–
Krawczyk (2017)	1. Buprenorphine 2. Methadone	1. Odds of OAT receipt	1. Race/ethnicity	1. Compared to white clients, the odds of receiving OAT were greater for Black clients (aOR = 1.37, 95% CI 1.24, 1.52) and greater for Hispanic clients (aOR = 1.21, 95% CI 1.11, 1.32) 2. These results varied significantly by reason for admission (heroin or other opioids)
Krawczyk (2022)	1. MOUD only 2. MOUD&SSP 3. SSP only	Comparison of patients across 3 types of services	1. Gender 2. Age 3. Race/ethnicity 4. Employment	1. A larger proportion of SSP only clients were female relative to MOUD only and MOUD&SSP (51.16% vs. 46.13% and 46.81% respectively; *p* < 0.001) 2. A larger proportion of MOUD only clients were between the ages of 41–60 compared to MOUD&SSP and SSP only (58.37% vs. 32.98% and 41.28%, *p* = 0.04) 3. Most clients in MOUD&SSP were either non-Latinx Black (29.72%) or Latinx (64.89%), but among MOUD-only and SSP-only clients, non-Latinx White had the largest prevalence (41.98% and 63.74%, respectively) (*p* < 0.001) 4. While the prevalence of unemployed participants was lower in the MOUD-only program compared to the combined and SSP-only programs (39.59% vs. 47,87% and 47,67%), more MOUD only clients reported being unable to work due to health compared to MOUD&SSP and SSP only (37.55% vs. 26.60%, 29.65%, *p* < 0.001)
Leston (2020)	1. Multidisciplinary healthcare team 2. SSP 3. Medication assisted treatment	Initiation/Access to treatment	1. Geospatial location	1. Participants described lack of access to Medication Assisted Therapy (MAT) due to geographical scarcity: “*And a lot of doctors weren’t even (prescribing MAT) … Most of them that I called weren’t taking any new patients. And then I just kept calling and calling until I found one that would take me, you know? It’s a four-hour drive once a month…I go to Cheyenne, Wyoming, once a month to do it, to get my prescription filled.*” (Participant B) 2. Participants felt that the quality of harm reduction services were varied in availability and quality by geographic location: “*I would like to see more treatment centers. I'd like to see more outpatient treatment centers.*” (Participant G)
Martinez (2011)	1. Methadone 2. Outpatient treatment 3. Residential treatment 4. Self-help	Odds of receiving drug treatment (all four services combined) in the past 6 months	1. Race/ethnicity	1. Mexican IDUs had a smaller prevalence of 100% syringe coverage—having a large enough supply of sterile syringes to meet demand—compared to White and African American IDUs (40%, 47%, 52% respectively, *p* < 0.01) 2. Mexican American IDUs had higher odds of using drug treatment in the past 6 months than White IDUs (aOR = 1.6, 95% CI 1.1, 2.1)
Nolen (2023)	1. Naloxone	1. Rate of receiving naloxone 2. Geospatial analysis of New York City (NYC) neighborhoods	1. Race/ethnicity 2. Geospatial location	1. Compared to White residents, Black residents had a higher naloxone receipt rate (aRR = 2.10, 95% CI 1.54, 2.85) 2. Compared to White residents, residents of other race (not Latino, Black, or White) had a lower naloxone receipt rate (aRR = 0.79, 95% CI 0.67, 0.92) 3. Northern and northwestern neighborhoods in NYC had higher than expected naloxone receipt rates (“hot spots”) for all racial/ethnic groups 4. In predominantly Latino and non-Latino Black neighborhoods in NYC, there were hot spots for Latino, non-Latino Other, and non-Latino White residents 5. There were hot spots for non-Latino Black residents in predominantly non-Latino White neighborhoods of NYC 6. Southern and southeastern areas of NYC demonstrated lower-than-expected rates of naloxone receipt (“cold spots”) only for non-Latino Black and Latino groups
Pro (2022)	1. Outpatient treatment	1. Positive treatment response, defined as reduction in use between admission and discharge	1. Housing 2. Race/ethnicity	1. Previously homeless clients who transitioned to independent living had over 3 times higher odds of positive treatment response compared to those who remained homeless (aOR = 3.78, 95% CI 3.49, 4.10) 2. Black clients had lower odds of positive treatment response than White clients (aOR = 0.82, 95% CI 0.73, 0.91) 3. AI/AN clients had higher odds of positive treatment response than White clients (aOR = 1.90, 95% CI 1.41, 2.54) 4. Hispanic clients had higher odds of treatment response than White clients (aOR = 1.45, 95% CI 1.32, 1.59) 5. Transitioning from homelessness to dependent housing was significantly associated with positive treatment response among White clients (aOR = 3.57, 95% CI 3.15, 4.06), Black clients (aOR = 1.79, 95% CI 1.41, 2.27), and Hispanic clients (aOR = 2.11, 95% CI 1.55, 2.86) 6. Transitioning from homelessness to independent living was significantly associated with positive treatment response among White clients (aOR = 4.70, 95% CI 4.26, 5.19), Black clients (aOR = 3.01, 95% CI 2.44, 3.71), and Hispanic clients (aOR = 2.07, 95% CI 1.74, 2.49)
Reynolds (2006)	1. Primary care 2. ER visit	1. Use of Primary healthcare provider (HCP) services 2. Use of ER services	1. Income 2. Employment 3. Race/ethnicity	Primary HCP 1. Reporting disability income was associated with increased odds of using primary HCP (OR = 5.04, 95% CI 2.2, 11.4) 2. Having income from welfare was associated with increased odds of using primary HCP (OR = 2.63, 95% CI 1.7, 3.9) 3. Reporting income from SSI (Supplemental Security Income) was associated with lower odds of accessing primary HCP (OR = 0.30, 95% CI 0.11, 0.77) ER Services 1. Participants reporting income from welfare had increased odds of using ER services (OR = 2.01, 95% CI 1.34, 3.03) 3. Individuals who were currently employed had increased odds of using ER services (OR = 1.82, 95% CI 1.20, 2.77) 4. Receiving income from spouse/family/friend was associated with increased odds of using ER services (OR = 1.57, 955 CI 1.06, 2.31) 5. African American race was associated with decreased odds of using ER services (OR = 0.31, 95% CI 0.18, 0.54)
Salow (2023)	1. SSP	1. Comparison of SSP program demographics vs general PWHID population in Seattle	1. Race/ethnicity 2. Age 3. Geospatial location 4. Housing	1. A larger proportion of participants in SSP were younger, ages 18–29 (23.1%), 30–39 (39.1%) compared to general PWHID population (17.5% and 29.6% respectively, *p* < 0.001) 2. There were less AI/AN individuals in SSP than general PWHID population (11.7% vs. 23.4%, *p* < 0.001) 3. There were more Asian/South Asian individuals in SSP than the general PWHID population (5.4% vs. 2.6%, *p* = 0.031) 4. There were less Black/African American SSP participants than the general PWHID population (6.0% vs. 19.6%, *p* < 0.001) 4. There were less Latinx/Hispanic SSP participants than the general PWHID population (7.3% vs. 12.8%, *p* = 0.009) 5. There were less Native Hawaiian/Pacific Islander SSP participants than the general PWHID population (1.9% vs. 4.5%, *p* = 0.034) 6. There were more White SSP participants than the general PWHID population (78.0% vs. 71.6%, *p* = 0.030) 7. There were less individuals currently experiencing homelessness in the SSP program than the general PWHID population (48.6% vs. 60.3%, *p* = 0.001) 8. A larger proportion of SSP clients lived in North Seattle (16.6% vs. 13.6%) and South King County (20.4% vs. 6.6%) compared to general PWHID population, and a smaller proportion of SSP clients lived in downtown (35.1% vs. 44.1%) and South Seattle (9.5% vs. 22.8%, *p* < 0.001)
Shrestha (2024)	1. Methadone 2. Buprenorphine 3. Naloxone	1. Predictors of methadone and buprenorphine treatment	1. Race/ethnicity	1. Less Latinx patients reported access to naloxone compared to non-Latinx patients (79.2% vs 88.3%, *p* = 0.02) 2. Less Latinx patients were currently using buprenorphine treatment compared to non-Latinx patients (11.8% vs. 21.5%, *p* = 0.026) 3. Identifying as Latinx was associated with less harm reduction facilitators (β = −0.49, *p* < 0.05) 4. Identifying as Latinx was positively associated with methadone treatment (β = 0.89, *p* < 0.05)
VanderWaal (2001)	1. Methadone 2. Syringe distribution 3. Needle cleaning supplies 4. National prevention campaigns 5. Mass-media prevention campaigns	1. Access to treatment	1. Geographical disparities and policies	1. Patients identified barriers to methadone clinics, including that their community did not have a clinic 2. Patients also described that a needle distribution program was not allowed by the city government in their community
Williams (2010)	1. Syringe distribution	1. Likelihood of using non-SEP sources for syringe access	1. Geographical disparities 2. Race/ethnicity	Model 1 results: SEP-home distance 1. A one mile increase from the SEP and the patient’s house resulted in a 6% increased likelihood of using non-SEP sources for syringes (OR = 1.05, 95% CI 1.02, 1.10) 2. Black patients had higher odds of accessing syringes from non-SEP sources than White patients (OR = 1.65, 95% CI 1.22, 2.25) 3. Latino patients had higher odds of accessing syringes from non-SEP sources than White patients (OR = 1.57, 95% CI 1.03, 2.39) 4. Latino patients were more likely to access syringes from non-SEP sources than White patients Model 2 results: SEP-buy location 5. Increased distance from SEP and buy locations increased the odds of using non-SEP sources for syringes (OR = 1.57, 95% CI 1.25, 1.97) 6. Black patients had higher odds of accessing syringes from non-SEP sources than White patients (OR = 2.08, 95% CI 1.48, 2.92) 7. Increased distance from SEP and buy locations among Latino IDU had over 6 times the odds of using syringes from non-SEP sources (OR = 6.70; 95% CI 2.32, 19.4) Model 3 results: SEP-use location 8. Black patients had higher odds of accessing syringes from non-SEP sources than White patients (OR = 1.84, 95% CI 1.39, 2.44) 9. Increased distance from SEP and use locations among Latino IDU had much higher odds of using non-SEP sources for syringes (OR = 5.35; 95% CI 2.53, 11.3)

aD = adjusted difference; CI = confidence interval; IDU = injection drug use; PWHID = people with history of injecting drugs; SSP = sterile syringe program; aOR = adjusted odds ratio; MOUD = medication for opioid use disorder; ER = emergency room; OAT = opioid agonist treatment; AI/AN = American Indian/Alaskan Native; SEP = syringe exchange program. Factors with ( +) indicate a positive association with positive health outcomes, and (-) indicate a positive association with negative health outcomes

### Racial and ethnic minority groups

The association between race/ethnicity and service utilization was highly inconsistent across studies. Several studies found that participants of non-White race had more positive outcomes compared to White participants across all of their respective outcomes of interest, including ancillary services, MOUD, drug treatment service usage, and sterile syringes [44, 56, 58, 65]. On the other hand, other studies that investigated race/ethnicity as a factor related to harm reduction service usage found that participants of White race observed higher rates of positive outcomes compared to non-White participants, regardless of the outcome of interest [48, 50, 52, 61]. An additional four studies showed that sometimes White participants had better outcomes and sometimes non-White participants did, depending on the outcome [11, 46, 59, 60].

Other studies compared different racial/ethnic groupings. One study found that Latinx participants had higher odds of using drug treatment programs and ERs compared to Black participants [53]. Another study found that Latinx patients had less access to naloxone, buprenorphine, and harm reduction facilitators compared to non-Latinx patients—although identifying as Latinx was positively associated with methadone treatment [63].

In other studies, the relationship between race/ethnicity and harm reduction services was less clear. For example, one article comparing clients in MOUD-only treatment, MOUD and sterile syringe program (SSP) treatment, and SSP-only treatment found that MOUD-only and SSP-only clients were mostly non-Latinx White, while clients in the other combined program were largely non-Latinx Black or Latinx participants [55]. A study comparing PWHID in an SSP program compared to PWHID in King County, WA, found that a larger proportion of SSP clients were Asian/South Asian, while there were fewer American Indian/Alaskan Native People (AI/AN), Black, Latinx, and Native Hawaiian or Other Pacific Islander (NH/PI), than the general population [62].

### Age

Three studies investigated age as a factor contributing to harm reduction service initiation and adherence; the association between age and service use appeared to be dependent on certain service types. Older age was significantly associated with higher odds of carrying naloxone in one study [52]. A negative relationship between older age and SSP usage was also observed; older age was associated with being in a MOUD-only program as opposed to a combined MOUD and SSP program or an SSP-only program in one study [55], and was associated with not being in an SSP in another study [62].

### Gender and sexual orientation

Most studies that investigated gender and service usage found that female PWHID had higher service utilization than males. One study found a positive association between identifying as female and carrying/accessing naloxone compared to males [52]. Another study found that people in a high injection and sexual risk group had significantly more women and higher naloxone utilization, although the direct association between gender and naloxone was not assessed [47]. Participants in SSP-only programs were more likely to be female, while participants in MOUD-only and combined MOUD and SSP programs were more likely to be male [55]. One study found an inverse association between entering methadone maintenance treatment among males and females of different races and ethnicities; Chicano males more often reported entering treatment due to legal coercion compared to Anglo males, while the opposite was true for Chicano and Anglo females [46].

### Geographic location

Geographic location was found to be a significant factor related to harm reduction service initiation and adherence in six different studies [48, 57, 59, 62, 64, 65]. Participants recruited from specific areas (e.g., Raleigh, NC, northern and northwestern New York City [NYC], North Seattle, and South King County) had higher odds of using certain services such as sterile syringes and naloxone [48, 59, 62]. While three studies highlighted how geographical isolation contributed negatively to accessing harm reduction services due to a lack of transportation and scarcity of services such as SSPs and methadone clinics [57, 64, 65], no studies directly compared service initiation or adherence across urban, suburban, and rural settings.

### Socioeconomic status

Several studies investigated factors related to socioeconomic status, including employment, insurance, education levels, and housing status. A comparison of patients who attended MOUD-only programs, combined MOUD and SSP programs, and SSP-only programs found that participants who attended MOUD-only programs had fewer unemployed participants than the other programs, but a higher prevalence of those being unable to work due to health [55]. People who reported receiving income from disability or welfare were associated with higher odds of using primary care, while income from Supplemental Security Income was associated with lower odds of using primary care [61]. This same study showed that income from welfare, being currently employed, and receiving income from a spouse/family/friend were associated with higher odds of using ER services [61]. One study found that being a Medicaid recipient was associated with higher odds of using harm reduction services than those who were not insured by Medicaid [44]. On the other hand, another study found that using insurance coverage to pay for services was associated with longer admission delays when compared to self-payment [50].

There was a clear positive association between education and service usage. In one study, participants who completed high school had significantly lower odds of admission delays to methadone outpatient treatment compared to those who had not completed high school [50]. Another study found that participants with some college education had over double the odds of carrying naloxone compared to lower education levels [52].

The studies that assessed housing status and service initiation and adherence found conflicting evidence. One study reported that a smaller proportion of participants of an SSP were people experiencing homelessness compared to the general PWHID population [52]. Another study found that participants who transitioned from homelessness to stable housing—particularly independent living—had better outpatient treatment outcomes (a reduction in substance use) compared to participants who remained homeless [60]. On the other hand, one study observed that those experiencing unstable housing had higher odds of being in a latent class that was linked to higher SSP visits and naloxone access [47], and another found homelessness significantly associated with higher odds of often/always carrying naloxone [52].

### Initiation and adherence to harm reduction services

Factors significantly associated with initiation of and adherence to harm reduction services were categorized into negative or positive associations and subsequently sorted into factors related to the individual seeking the services or factors related to the service itself. The extracted findings related to these factors are discussed below and presented in Table 3.

**Table 3:** Factors associated with harm reduction service initiation and adherence (n = 25)

First author (Year)	services	Outcome	Factors associated with outcome*	Findings
Anastario (2017)	1. Sterile syringes	1. Obtaining new syringes	1. Knowledge ( +)	1. Knowledge of hepatitis C was positively correlated with the reported level of ease in obtaining new syringes (rho = 0.23, *p* = 0.099) 2. Knowledge of HIV was positively correlated with the reported level of ease in obtaining new syringes (rho = .25, *p* = 0.071)
Barnett (2023)	1. Hospitalization 2. Outpatient care 3. Emergency room care 4. Buprenorphine 5. Naloxone 6. Benzodiazepine	1. Health care utilization 180 days after index events	–	–
Bass (2022)	1. Drug test strips	1. Using fentanyl drug testing strips before IDU	1. Lack of patient knowledge (-) 2. Low perceived need for service (-) 3. Inconvenience (-) 4. Severity of addiction (-)	1. Of the 20 participants interviewed, only 20% were interested in or had experience using fentanyl test strips including knowing where to obtain them and using them as a safety measure 2. Despite positive beliefs about fentanyl test strips (e.g., the test strips could help them *"be more safer”*) and even owning test strips, participants did not often use them 3. Participants felt they were not needed because they could identify fentanyl by smell, the test strips took too long (*“as soon as I get the bag of dope, I want to snort that before the cops come”*), or they needed fentanyl more (*“They’re good to have. But at this point, I need fentanyl”*)
Beidelman (2023)	1. Supporting services 2. Maintenance 3. Primary care	1. Odds of using ancillary services - Cluster 1: no/low use - Cluster 2: high use of support, maintenance, and primary care - Cluster 3: high use of all	1. Living alone (-) 2. Longer length of being in treatment ( +) 3. High number of service contacts ( +)	1. PWHID who live alone had lower odds (aOR = 0.08, 95% CI: 0.04, 0.17) of being in Cluster 2 (high use of some services) than Cluster 1 (no/low use of services) 2. PWHID who live alone also had lower odds (aOR = 0.16, 95% CI: 0.06, 0.44) of being in Cluster 3 (high use of all services) than Cluster 1 (no/low use of services) 3. Participants that had been clients for a longer period had higher odds (aOR = 1.27, 95% CI: 1.01, 1.61) of being in Cluster 2 (high use of some services) than Cluster 1 (no/low use of services) 4. For each additional service contact a participant had, they had higher odds of being in Cluster 2 (high use of some services) (aOR = 18.34, 95% CI: 6.73, 49.97) and Cluster 3 (high use of all services) (aOR = 18.19, 95% CI: 6.68, 49.57) compared to Cluster 1 (no/low use of services)
Bonar (2014)	1. Test shots 2. Skin cleaning before injection	1. Using test shots prior to IDU 2. Skin cleaning prior to IDU	1. Inconvenience (-) 2. Lack of patient knowledge (-) 3. Lack of treatment supplies (-) 4. Being in withdrawal (-) 5. Not wanting to waste drugs (-)	1. For participants that did not clean their skin before injecting, 40% reported that it was because it took too much time 2. Likewise, 24% of participants said they did not do test shots because they were rushing, hurrying or had time concerns 3. Several participants reported not cleaning their skin (23%) or doing test shots (13%) because they were in withdrawal 4. Participants did not clean their skin prior to injection because it did not occur to them (19%), and a similar (14%) said the same for not occurring to them to do test shots 5. Participants did not do test shots because they are familiar with batch or dealer and knew the strength already (20%) 6. Skin cleaning was not done because they did not have access to supplies (19%) 7. Some participants didn’t do test shots due to not enough drugs/waste of drugs /not enough to get high (15%)
Brecht (1993)	1. Methadone	1. Reason for entering methadone maintenance (MM) treatment 2. Variables associated with treatment effectiveness	1. Legal coercion 2. Non-legal reasons for entering	Reason for Entering – Treatment Outcomes 1. Percent time of no narcotics use increased significantly over time and had a significant three-way interaction between ethnicity, legal coercion, and time (*p* < 0.01). All legal coercion groups increased in percent of time with no narcotics use over time, but there were higher relapse rates after treatment completion for Chicanos (both male and female) in moderate (62% during treatment to 32% posttreatment) and high (27% to 17%) legal coercion groups. There was a decrease in narcotic abstinence among Anglo participants with high legal coercion (41% to 39%), Chicano participants under low legal coercion (35% to 30%). There was a continued increase in percent time no narcotics use among Anglo participants with moderate coercion (36% to 45%) and Anglo participants with low legal coercion (41% to 44%) 2. MM treatment on their first admission slightly longer (average 23 months) than those under less severe legal coercion (average 19 months) or voluntary admissions (average 20 months)
Chueng (2022)	1. SSP 2. Naloxone	1. Odds of SSP visits and naloxone utilization by risk class: - LIHS: Low Injection, High Sexual risk - HIMS: High Injection, Moderate Sexual risk - LILS: Low Injection, Low Sexual risk	1. Methamphetamine use 2. Heroin use (-) 3. Speedball use (-)	1. Participants who reported methamphetamine injection had higher odds of being in LIHS than LILS (aOR = 4.35, 95% CI 1.97, 9.60) 2. Those who reported methamphetamine injection had higher odds of being in LIHS than HIMS (aOR = 4.35, 95% CI 1.97, 9.60) 3. Participants who reported heroin injection had higher odds of being in HIMS than LILS (aOR = 2.76, 95% CI 1.60, 4.78) 4. Participants who reported speedball injection had higher odds of being in HIMS than LILS (aOR = 2.36, 95% CI 1.54, 3.61)
Costenbader (2010)	1. Syringe distribution	1. Odds of pharmacy as main source of syringe	1. Co-occurring HCV ( +) 2. IDU frequency ( +) 3. Risk of IDU in past 30 days (-)	1. Testing positive for HCV was associated with higher odds (aOR 2.04, 95% CI 1.45–2.87) of having a pharmacy be the primary source of syringe purchases 2. Injecting 30 or more times was associated with higher odds (aOR 1.56, 95% CI 1.06–2.30) of having a pharmacy be the primary source of syringe purchases 3. Any risk of IDU in the past 30 days was associated with lower odds (aOR 0.47, 95% 0.32–0.69) of having a pharmacy be the primary source of syringe purchases
Gryczynski (2011)	1. Methadone	1. Odds of delay in admission to outpatient methadone treatment	1. Referral source 2. Previous IDU treatment (-) 3. IDU ( +) 4. Alcohol use (-) 4. Recurring psychiatric condition (-) 5. Community treatment system utilization	1. There were lower odds of admission delays among participants referred by substance use providers (OR = 0.478, 95% CI 0.422, 0.542) and health care providers (OR = 0.362, 95% 0.301, 0.435) compared to self-referrals 2. Participants referred to outpatient methadone treatment by the criminal justice system were more likely to experience delay in admission compared to self-referrals (OR = 1.698, 95% CI 1.445, 1.996) 3. Participants with a higher number of previous treatment episodes for substance use was associated with admission delay (OR = 1.051, 95% CI 1.030, 1.072) 4. Participants who reported IDU had lower odds of delay (OR = 0.917, 95% CI 0.854, 0.983) 5. There were higher odds of admission delay among participants who reported using alcohol (OR = 1.226, 95% CI 1.102, 1.364) and cocaine (OR = 1.115, 95% CI 1.038–1.198) 6. Participants with recurring psychiatric problems had higher odds of admission delay (OR = 1.197, 95% CI 1.092, 1.313) 7. Higher utilization of community outpatient methadone services was associated with higher odds of admission delays (OR = 1.006, 95% CI 1.001, 1.012) 8. Higher utilization of community services of alternative modalities had lower odds of admission delays (OR = 0.995, 95% CI 0.992, 0.999)
Gryczynski (2013)	1. Buprenorphine 2. Methadone	1. Reasons for using buprenorphine instead of methadone 2. Reasons for not using methadone	1. Patient readiness ( +) 2. Perceived medication effectiveness ( +) 3. Stigma (-) 2. Medication side effects (-) 3. Negative prior experiences (-)	1. Personal readiness for treatment or recovery was offered by 58.8% of the sample. The theme of treatment readiness was generic in the sense that it could be applied to seeking any kind of addiction treatment and not buprenorphine specifically 2. Withdrawal suppression was cited as a reason for choosing buprenorphine by 32.5% of the sample 3. Participants indicated that choosing buprenorphine would help them reach a state of "normalcy" that was not achievable with heroin or methadone due to buprenorphine’s non-sedating effects (25.0%) 4. More than half (52.5%) of participants indicated that they chose buprenorphine as a better alternative to methadone. Methadone was perceived as an inferior alternative that had limited benefits, had worse side effects, that methadone was a long-term treatment while buprenorphine was short-term, and that methadone was a harmful substance while buprenorphine was a medicine *(“I see people do the methadone and it's nasty and it puts me to sleep…. Taking the buprenorphine is like taking a vitamin, so it's strengthening me.”*). Nearly all (91.3%) participants reported some negative characteristic of methadone 5. Participants reported learning about buprenorphine from various different non-medical sources, including friends and family (12.5%), while only a few participants (7.5%) had personal experience with methadone. Many (27.5%) learned about buprenorphine from non-medical sources 6. Participants reported choosing buprenorphine over methadone due to perceptions that methadone has worse pharmacological and health outcomes 7. Participants responses also indicated stigma against methadone
Heidari (2024)	1. Outpatient treatment 2. MOUD 3. ER visits	Engagement and satisfaction with services	1. Ease of navigation ( +) 2. Co-occurring psychiatric condition ( +) 3. Positive relationship with healthcare provider ( +) 4. Integrated services ( +) 5. Perceived and diagnosed needs-alignment ( +) 6. Social reinforcement ( +) 7. Stigma (-)	1. Healthcare access and wrap-around services helped patients navigate non-medical needs (e.g., health insurance navigation, reduced financial burden, filing disability, supplemental nutrition assistance and linkage to housing) which, in turn, facilitated engagement in medical care 2. Positive, friendly patient-provider relationships helped participants view their healthcare experiences positively and was key element in desire to remain engaged more in care 3. Participants felt that integrated care services helped facilitate care engagement. Participants with cooccurring mental health diagnosis further emphasized an appreciation for when mental health and primary care were integrated 4. Engagement in and satisfaction with care was higher for participants whose self‐perceived needs aligned with the actual needs diagnosed by their provider 5. Having social network support was a powerful means through which some participants felt encouraged to remain engaged in recovery and chronic disease treatment 6. While most participants reported using MOUDs to support healthcare engagement through reducing abstaining from drug use, some had negative sentiments towards MOUDs compared to detox and group counseling recovery methods and felt that MOUDs were “trading one drug for another.” 7. Patients felt better equipped to engage in healthcare to reduce substance use when their providers were supportive, non-judgmental, and offered resources
Jones (2023)	1. Naloxone	Odds of always/often carrying naloxone	–	–
Kang (2006)	1. Methadone 2. Outpatient programs 3. Residential treatment 4. ER visits	1. Odds of any drug treatment enrollment 2. Comparison of enrollment in drug treatment and ER visits between African American and Hispanic patients	1. Crack cocaine use (-) 2. IDU ( +)	1. Among African Americans, participants who used crack cocaine had significantly lower odds of enrolling in drug treatment (aOR = 0.28, 95% CI 0.09, 0.86) 2. African American participants who have ever injected drugs had significantly higher odds of enrolling in drug treatment (OR = 19.44, 95% CI 6.11, 61.89)
Kim (2015)	1. Sterile syringes	1. Likelihood of using new sterile needles	1. Source of needles	1. PWHID who reported getting needles from a friend had lower odds of always using a sterile syringe with each injection in 2005 (OR = 0.31, 95% CI 0.20, 0.48), 2009 (OR = 0.38, 95% CI 0.25, 0.59), and 2012 (OR = 0.55, 95% CI 0.37, 0.82) 2. Likewise, PWHID who reported getting needles from a dealer had lower odds of always using a sterile syringe with each injection in 2005 (OR = 0.49, 95% CI 0.33, 0.72), 2009 (OR = 0.50, 95% CI 0.33, 0.76), and 2012 (OR = 0.43, 95% CI 0.29, 0.64) 3. Getting needles from pharmacies was associated with lower odds of always using a sterile needle in 2009 (OR = 0.53, 95% CI 0.36, 0.77) and in 2012 (OR = 0.67, 95% CI 0.46, 0.98)
Krawczyk (2017)	1. Buprenorphine 2. Methadone	1. Odds of OAT receipt	–	–
Krawczyk (2022)	1. MOUD only 2. MOUD&SSP 3. SSP only	Comparison of patients across 3 types of services	1. Drug use history 2. OD history 3. History of sharing injection equipment 4. History of injection equipment confiscation 5. Past substance use treatment	1. More SSP only clients (94.77%) reported using any drugs in the past month than MOUD only (83.27%) and MOUD&SSP clients (85.11%, *p* = 0.02) 2. Less MOUD only clients (37.55%) reported using opioids in the past month than MOUD&SSP (65.96%) and SSP only clients (69.19%, *p* = 0.02) 3. More SSP only clients (91.86%) reported using any drugs in the past week than MOUD only (73.06%) and MOUD&SSP (80.85%, *p* < 0.001) 4. Less MOUD only clients (27.35%) reported using opioids in the past week than MOUD&SSP only (58.51%) and MOUD&SSP (61.63%, *p* = 0.003) 5. A larger proportion of participants in SSP only reported injecting any drugs in the past week than MOUD only and MOUD&SSP (67.44%, 51.84%, 56.38% respectively, *p* < 0.001) 6. The largest proportion of people who OD in the past month attended SSP only (16.28%), followed by MOUD&SSP (10.64%) and MOUD only (4.49%, *p* = 0.003) 7. Many more SSP only clients went to a syringe exchange in the past month than MOUD only and MOUD&SSP (63.37%, 8.16%, 51.06% respectively, *p* < 0.001) 8. More SSP only clients (11.05%) gave someone a syringe after using it in the past month compared to MOUD only (2.04%) and MOUD&SSP clients (0%, *p* < 0.001) 9. More SSP only clients (11.63%) used a syringe after someone used it in the past month compared to MOUD only (1.22%) and MOUD&SSP clients (0%, *p* = 0.002) 10. More SSP only clients (21.51%) used cookers/rinse water after someone used it in the past month compared to MOUD only (2.04%) and MOUD&SSP clients (6.38%, *p* < 0.001) 10. More SSP only clients (22.09%) gave cookers/rinse water after using it in the past month compared to MOUD only (3.67%) and MOUD&SSP clients (4.26%, *p* < 0.001) 11. Syringes being confiscated by the police in the past month was more common among SSP only clients (9.88%) compared to MOUD only (1.63%) and MOUD&SSP clients (5.32%, *p* < 0.001) 12. Almost all MOUD only clients (98.37%) reported receiving any substance use treatment in the past month, compared to less prevalence among MOUD&SSP clients (34.04%) and SSP only clients (41.28%, *p* < 0.001) 13. More MOUD only clients reported individual counseling in the past month than MOUD&SSP and SSP only clients (92.65%, 26.60%, 30.23% respectively, *p* < 0.001) 13. More MOUD only clients reported group counseling in the past month than MOUD&SSP and SSP only clients (51.43%, 8.51%, 9.88% respectively, *p* = 0.0002) 14. Attending self-help groups in the past month was reported by a larger proportion of MOUD only clients (28.57%) compared to MOUD&SSP (12.77%) and SSP only (8.72%, *p* < 0.001) 15. More MOUD only clients reported receiving methadone for opioid addiction in the past month compared to MOUD&SSP and SSP only clients (91.84%, 15.96%, and 21.51%, *p* < 0.001)
Leston (2020)	1. Multidisciplinary healthcare team 2. SSP 3. Medication assisted treatment	Initiation/Access to treatment	1. Patient motivation and self-efficacy ( +) 2. Social and community stigma (-) 3. Stigma from healthcare providers (-) 4. Lack of health provider knowledge (-) 5. Negative prior experiences (-) 6. Cultural policies/stigma (-)	1. Participants reported that feeling stigma, shame, guilt, judgement, and fear of negative interactions—primarily from healthcare providers and local communities—prevented PWHID from pursuing treatment: *“Once you get labelled an IV drug user, your name's just nothing. So I mean it…Because I remember whenever I wasn't one, you know. I remember whenever I didn't have that stigma. Doctors treated me so well, greeted me so nicely, you know. And now doctors look at me like, what's wrong with you? Nothing's wrong with you.”* 2. Participants described lack of knowledge and access to medication assisted treatment (MAT) by both providers (*“Well first of all, they don’t support them because they're not, like, offering clean needles.”*) and PWHID (including being reluctant to give out personal information in exchange for services) as a barrier to initiation 3. There was a lack of substance use services where providers had compassion: *“I think they should be more, it seems like they're more of just there, mainly the counselors that do work at treatment, whatever treatment they're working at, is just work for them, another job, you know. Kind of lost their enthusiasm about what they're really, why they really wanted to be a counselor.”* 4. Participants reported that having access to safe, unused syringes was associated with risk-taking behaviors (e.g., sharing, unhygienic needle cleaning, exchange sex) was common: *“Oh, trade in, like, sex. I mean, they'll trade that for the fix or whatever.”* 5. Participants reported that feeling motivated and feeling self-efficacy to succeed in treatment were encouraging factors in pursuing treatment. *“It only works if you want it to work. And if you’re just doing it because the court wants you to, more than likely, you’re going to fall back to using again after treatment.”* 6. While some participants felt that tribal council provided adequate health services for PWHID, other participants felt that tribal policies towards drug use were aggressive, strict, and made PWHID feel isolated: *“I think the Tribal Council is trying to help people. I just think they’re in the beginning process of it and I think like any process it takes time.”*
Martinez (2011)	1. Methadone 2. Outpatient treatment 3. Residential treatment 4. Self-help	Odds of receiving drug treatment (all four services combined) in the past 6 months	–	–
Nolen (2023)	1. Naloxone	1. Rate of receiving naloxone 2. Geospatial analysis of New York City (NYC) neighborhoods	–	–
Pro (2022)	1. Outpatient treatment	1. Positive treatment response, defined as reduction in use between admission and discharge	–	–
Reynolds (2006)	1. Primary care 2. ER visit	1. Use of primary healthcare provider (HCP) services 2. Use of ER services	1. Chlamydia diagnosis ( +) 2. Drug use history ( +) 3. Hepatitis B diagnosis ( +)	1. Ever having had chlamydia increased odds of using primary HCP (OR = 2.69, 95% CI 1.6, 4.5) 2. Number of days in the last 30 used other opiates other than heroin increased odds of using primary HCP (OR = 1.04, 95% CI 1.002, 1.1) 3. Ever having hepatitis B was associated with increased odds of using ER services (OR = 2.12, 95% CI 1.33, 3.36) 4. Number of days in the last 30 used opiates other than heroin was associated with increased odds of using ER services (OR = 1.06, 95% CI 1.02, 1.09)
Salow (2023)	1. SSP	1. Comparison of SSP program demographics with general PWHID population in Seattle	1. Higher IDU frequency ( +)	1. Less SSP survey participants reported injecting drugs daily (67.5%) compared to 85.3% of the general PWHID population reported injecting drugs daily (*p* < 0.001)
Shrestha (2024)	1. Methadone 2. Buprenorphine 3. Naloxone	1. Predictors of methadone and buprenorphine treatment	–	–
VanderWaal (2001)	1. Methadone 2. Syringe distribution 3. Needle cleaning supplies 4. National prevention campaigns 5. Mass-media prevention campaigns	1. Access to treatment	1. Government and media distrust (-) 2. Lack of patient knowledge (-) 3. Lack of provider understanding (-) 5. Negative stigma towards medication (-) 6. Cost of treatment (-) 7. Lack of transportation (-) 8. Low perceived treatment effectiveness (-) 9. Stigma towards patients (-)	1. Participants reported that written harm reduction campaigns would be unrealistic given health literacy levels and distrust in the government: *“It [information] will get out, but only to a select few, because not everybody can read.”* 2. Participants felt that professional services were out of touch with communities and many did not trust them, with rare exceptions: *“A professional ain’t going to take the time to come and talk about it [prevention]. He might come to study us, the culture, the drug users, and prostitutes…you feel like a bug, instead of a person. He comes to get a grade and some recognition of stuff that he be doing.”* 3. Participants reported a lack of readily available methadone clinics, citing transportation barriers among IDUs: *“I would say nine out of ten [have] no transportation.”* 4. Participants did not have knowledge about referral to methadone clinics 5. Participants felt that using methadone was another form of drug dependence 6. Participants felt that regularly attending methadone clinics was too costly 7. Participants argued that successful harm reduction services should be realistic and not expect abstinence from drugs:* “When you go to treatment, they say you got to totally stop drinking, you got to totally stop using drugs. That’s not going to happen in this community. In the treatment programs here, they say you either totally quit or you’re a failure. Nobody likes to be classified a failure.”* 8. Many participants endorsed the idea of distribution of sterile syringes and felt that needle cleaning supplies were less effective: *“We need the availability of the needles program to make sure there is a supply of needles in around the neighborhood.”* 9. Some participants felt that the community, particularly the African American community, would be opposed to harm reduction efforts: *“The people in the community don’t want that [needle distribution] to happen because they don’t want it to look like everybody is using drugs. I think the community is against that.”* 10. Many participants felt that harm reduction efforts should be from within the IDU community and from providers with similar life experiences and demographics: *“The programs are good, but we need qualified people in the programs as well. When we get into the program, we need some people that know where we are coming from. Education is cool, don’t get me wrong, because I go to school, but we need some people that would know where I’m coming from.”* 11. Participants endorsed the idea of smaller, structured groups and other organized educational programs like community-based programs. *“Another thing that would work is a good educational program to train people how to work and take care of themselves. When they don’t have a lot of free time to explore drugs, it’s better for them.”* 12. Patients felt it was necessary to acknowledge race-based stigma by providers, and medical mistrust among the African American community:* “A lot of testing and counseling is done through the county health department, and it is predominantly Caucasian. This community is predominantly black. People just do not trust the workers there.”*
Williams (2010)	1. Syringe distribution	1. Likelihood of using non-SEP sources for syringe access	–	–

IDU = injection drug use; PWHID = people with history of injecting drugs; aOR = adjusted odds ratio; CI = confidence interval; SSP = sterile syringe program; MOUD = medication for opioid use disorder; ER = emergency room; OAT = opioid agonist treatment; AI/AN = American Indian/Alaskan Native; SEP = syringe exchange program. Factors with ( +) indicate a positive association with positive health outcomes, and (-) indicate a positive association with negative health outcomes

### Individual-level factors negatively associated with harm reduction services

Individual-level factors such as experienced stigma, past negative experiences, intrinsic patient qualities, and specific addiction and substance use behaviors played a large role in service utilization. The most reported individual-level factor was stigma (n = 5). Stigma towards injection drug use (IDU) or PWHID from interpersonal experiences, cultural policies, and medical providers impeded access and motivation to utilize harm reduction services [57, 64]. Other studies reported stigma from patients towards the harm reduction service itself, particularly MOUDs, wherein participants felt that taking a medication to reduce substance use was replacing one drug with another [49, 51, 64]. A history of negative experiences with the healthcare system, inadequate provider knowledge related to harm reduction services, and general distrust of healthcare, government, and media systems were all negatively associated with service initiation [49, 57, 64].

There were several intrinsic participant characteristics that repeatedly arose as a significant factor negatively associated with harm reduction services. Several studies found that lower patient knowledge, literacy, or awareness was negatively associated with the use of services such as fentanyl test strips among SSP participants [43], referrals to a methadone clinic or methadone itself [64], and, relatedly, not using test shots or skin cleaning before IDU because it did not occur to them to do so [45]. Participants in other studies reported that they felt that they didn’t need services [43], or that services wouldn’t be effective [64], which may be a reflection of not understanding the purpose or mechanism of harm reduction services.

Fear of potential side effects was another barrier to initiating MOUD use [49]. Additionally, one study found that having a co-occurring psychiatric condition was associated with a delay in admission to an outpatient methadone treatment [50]. Finally, one study found that living alone was negatively associated with using harm reduction services [44].

Finally, several studies found that specific substance use behaviors were negatively associated with initiating and adhering to certain harm reduction services. Individuals in two studies reported not using test strips, test shots, or cleaning skin before injecting drugs because they were in a rush, were in withdrawal, or did not want to waste drugs on test shots [43, 45]. Risk for recent IDU was negatively associated with sterile syringe use in North Carolina [48]. The use of specific substances had varying impacts on service initiation and usage. For example, there was a higher proportion of people belonging to a high injection and sexual risk group that reported heroin and speedball injection, and a lower proportion of SSP visits and naloxone utilization, although the association between drug type use and service use was not directly studied [47]. African American participants who used crack cocaine had lower odds of enrolling in outpatient and residential drug programs compared to those who did not use crack cocaine [53]. There were higher odds of delay to admittance to an outpatient methadone treatment program among people who reported alcohol use and being previously engaged in treatment for IDU [50].

### Individual-level factors positively associated with harm reduction services

There were several intrinsic participant characteristics positively associated with service initiation and adherence, including internal motivation, perceptions on treatment effectiveness, knowledge, previous diagnoses and healthcare experiences, and substance use behaviors. Several studies found that readiness, motivation, self-efficacy, needs aligning with the service, and reinforcement from social networks were positively associated with service usage [49, 51, 57]. Patient perceptions that MOUDs were highly effective were linked with initiation of MOUD treatment [49]. A study investigating factors associated with obtaining new sterile syringes found a positive correlation between knowledge about HCV and HIV with obtaining sterile syringes [42].

Co-morbidities including Hepatitis C [48], Hepatitis B [61], chlamydia [61], and psychiatric diagnoses [51], were positively associated with service use. One study found that a positive previous experience with healthcare or other treatment services was positively associated with initiation of new harm reduction services [51]. A study on outpatient methadone services found that clients who had previously used community methadone services and other resources had lower odds of admission delay [50]. Likewise, a study that compared participants across MOUD-only, MOUD and SSP, and SSP-only treatment options found that MOUD-only participants reported using opioids less than the other groups, and also reported higher odds of receiving prior IDU treatment, individual and group counseling, attending self-help groups, and receiving methadone compared to the other groups [55].

Multiple studies identified substance use behaviors positively associated with harm reduction service usage. PWHID who had been in treatment longer and had a higher number of service contacts had higher odds of using support services (e.g., employment, legal, transport, education), primary care, and maintenance services (e.g., case management, linkage to outside services, adherence to programs) [44]. Two studies found that a higher frequency of IDU was positively associated with using pharmacies to purchase new syringes and engaging in an SSP [48, 62]. Likewise, a study in Alaska found that increased frequency of using opiates aside from heroin was associated with increased odds of using primary care and ER services [61]. Participants who reported IDU had lower odds of admission delay to outpatient methadone treatment [50], and another study among African American patients living with HIV in NYC found that those who reported previously engaging in IDU had higher odds of enrolling in drug treatment programs. [53]

### Service-level factors negatively associated with harm reduction services

Factors attributable to the harm reduction service itself, such as service inconvenience, structural barriers, type of service, referral, and setting that were found to be negatively associated with service initiation. Several studies reported inconvenience as a barrier to service initiation; participants in two separate studies reported not using fentanyl test strips, test shots, or cleaning their skin before injecting because these services took too long [43, 45]. The latter study also reported not engaging in test shots or skin cleaning due to insufficient supplies [45]. Structural barriers, such as the cost of treatment and a lack of transportation to the service, were reported for methadone and syringe-related services [64]. One cross-sectional study in California found that patients who received syringes from a friend, dealer, or pharmacy had lower odds of always using a sterile syringe [54]. Participants who were referred to an outpatient methadone treatment by the criminal justice system had higher odds of admission delay compared to self-referral [50].

Service type also played a difference in harm reduction outcomes. A study comparing three types of programs found that, compared to clients of MOUD-only or combined MOUD and SSP programs, a higher proportion of SSP-only clients reported using drugs and injecting more frequently, overdosing, going to a syringe exchange, having injection equipment confiscated by police, and sharing injection equipment recently [55].

### Service-level factors positively associated with harm reduction services

The method of referral to harm reduction services was investigated by several studies, which have found a positive association with treatment initiation and adherence. One study found that entering a methadone maintenance program due to severe legal coercion was positively associated with increased time not using narcotics compared to other reasons for admittance [46]. Another study found that referrals to methadone treatment by substance use providers and healthcare providers had lower odds of admission delay compared to self-referrals [50]. Finally, one qualitative study among patients with multiple comorbidities reported that outpatient, ER, and MOUD services that were integrated with other healthcare services and were easy to navigate were positively associated with engagement and satisfaction [51].

## Discussion

This review identified various factors that were positively and negatively associated with the initiation and adherence to harm reduction services. More studies reported barriers to these services than facilitators. Disparities across non-intervenable patient characteristics such as race, age, and sexual orientation were present, albeit conflicting in some cases. Individual-level factors associated with using harm reduction services are largely centered around patient knowledge, stigma, past experiences, intrinsic motivation, and substance use behaviors. Service-level factors emphasized accessibility related to cost, geographical location, and source of referral.

Our findings aligned with the literature around barriers and facilitators to substance use disorder treatments, but there were some unique findings specifically related to harm reduction services for PWHID. Another literature review of factors associated with substance use disorder treatment around the world identified individual-level barriers (e.g., concerns about MOUDs, stigma, and intrinsic characteristics such as low motivation) and service-level barriers (e.g., long wait times and a lack of a qualified workforce) that align with ours [66]. Likewise, our facilitators largely aligned with individual-level factors (e.g., personal motivation, positive relationships with family and practitioners) [66]. Our review did not identify many service-level facilitators; however, wraparound services emerged as a facilitator in both reviews. Interestingly, our review did not find interpersonal barriers or facilitators (e.g., supportive network, negative role models, peer influence) to be highly prevalent with PWHID, suggesting that one’s social network may not be as influential when seeking or using harm reduction services related to IDU. Our review shows that there are unique barriers and facilitators for harm reduction services specifically for IDU compared to general substance use services.

Our study, conducted exclusively in the United States, largely aligned with a review assessing barriers and facilitators to harm reduction services among youth in British Columbia, Canada, which reported individual-level, interpersonal-level, and service-level factors. Individual-level barriers that aligned with our findings included stigma and discrimination, a lack of knowledge about available services, inaccessibility of services, scarcity of services due to geographic location, cost, and negative experiences with providers [67]. Like the international review for individuals with SUD, this review also found more interpersonal barriers and facilitators (e.g., supportive network) than our findings, and youth experienced more barriers related to their age and lack of autonomy [67]. In another global review that examined user involvement in substance use services, various factors were found that shaped their engagement with services, including being seen as a human, feeling validated, and addressing issues of stigma in interactions with substance use staff and professionals [68]. An overarching pattern in all three of these reviews is that there were far more barriers than facilitators; reducing these barriers will benefit people who use drugs around the world, especially through participatory and inclusive methods that challenge power imbalances and stigma.

In our review of studies, patient demographics, including age, race, and ethnicity, were often referred to as “barriers” to treatment initiation and adherence. However, this framing is problematic as immutable sociodemographic characteristics are not themselves barriers to harm reduction or services. For example, the use of race, a social construct, was often studied in biological terms to drive attitudes and beliefs about inherent differences between phenotypic groups [69]. Today, research uses race and ethnicity as a proxy measure for larger structural forces such as racism, which is problematic as it cannot adequately capture the process of racial stigma [70]. However, as more recent and contextualized research posits, the risk factor is racism, not race [71]. The same argument is applicable to other “barriers” discussed in the literature, including, but not limited to, education, gender, and sexual orientation. Future research should more accurately discuss sociodemographic characteristics in terms of disparities and inequities versus barriers or facilitators.

One factor associated with sociodemographic characteristics that may be intervened upon would be stable housing, which had a clear association with positive outcomes of harm reduction services in our study. With this finding, we argue that providing stable housing may be an intervention opportunity to reduce IDU. Housing costs have continued to rise in the US, unable to meet demand: an increasing number of American households are “cost-burdened”, meaning they spend more than 30% of their income on housing costs; in 2019, there were 37.1 million households that were cost-burdened [72], and in 2022, there were over 41 million – nearly a third of all households in the US [73]. Housing instability is a major social determinant of health, linked with lower access to healthcare and both adverse mental and physical health outcomes [74, 75]. Previous studies have shown that housing instability has negative associations with substance use, SUD, overdose, and death [76, 77]. Intervening on an upstream factor like housing may have substantial benefits for PWHID.

Results from this analysis suggest that combining harm reduction services with other healthcare services, or creating “wraparound” harm reduction efforts, may be beneficial to PWHID. Several studies found that prior usage of healthcare services [44, 50, 51], including those from co-morbidities that require treatment [48, 51, 61], were associated with positive harm reduction service outcomes. Providers also played a meaningful role in service usage, wherein a positive relationship with a provider was beneficial [51], but providers with stigma against PWHID and who lacked knowledge or understanding were detrimental [57, 64]. Other studies of patients with substance use disorders have reported that provider stigma and lack of preparedness were common reasons for not seeking harm reduction services [78]. As such, training providers about IDU and harm reduction and subsequently implementing harm reduction services or referral to services may be a future intervention strategy. For example, one pilot intervention study aims to address this barrier by implementing a universal patient screening, increasing education about harm reduction, and emphasizing referrals to community organizations from within the clinic [79]. A number of hospital-based harm reduction interventions have been shown to be effective and may be another beneficial method of implementing harm reduction services [80]. This strategy may also be beneficial for reducing transportation and geographic scarcity barriers that several studies reported [57, 64, 65]. Establishing standard harm reduction screening and services in primary care settings, for example, may normalize these services and reduce stigma against IDU, MOUDs, and other services. Stigma against IDU, PWHID, and MOUDs was the most common individual-level barrier identified in this review and would be a beneficial target for intervention.

Our study was not without limitations. Of the 25 studies included, 18 were cross-sectional, limiting our ability to draw longitudinal conclusions. Cross-sectional studies ultimately reduce the available data measuring the continuum of substance use response, as that requires following up with patients as they move through the cycle. Most studies in our review emphasized using MOUDs, syringe exchanges, and distribution as the primary health services, limiting available data on other services such as mental health services, outpatient visits, casework, and education. Further, our study focused solely on U.S.-based research, reducing the generalizability of our results. Although we did conduct a quality assessment, we ultimately did not choose to exclude any articles based on quality. Finally, literature reviews are beholden to the present research; future studies should address the gaps mentioned to improve the understanding of key factors related to service initiation and adherence, which ultimately influence treatment outcomes.

## Conclusion

This review identified several factors upon which future researchers may intervene to improve harm reduction service initiation and adherence. We also identified several characteristics of both patients (e.g., self-efficacy, social support) and treatment services (e.g., providing education) that facilitated service use and success that should be leveraged in future interventions. Notably, this review identified several patient characteristics (e.g., age, gender, race/ethnicity) that served as proxies for socioeconomic well-being and played a significant role in harm reduction success. Future research may find that engagement in harm reduction services increases when IDU screening and services are integrated into other healthcare services. This, in turn, may decrease stigma against IDU, PWHID, and the harm reduction services themselves.

## Supplementary Information

Additional file1.

## Data Availability

Not applicable; no original datasets were generated or analyzed in the current study and thus not available for sharing.
