# Correction: Barriers and facilitators to initiating and adhering to harm reduction services among people who inject drugs in the United States: a systematic review

**DOI:** 10.1186/s12954-026-01495-x

**Published:** 2026-06-30

**Authors:** Carrie L. Nacht, Britt Skaathun, Kristen Ogarrio, Reanna Durbin-Matrone, Laura Laura Wright, Rick Reich, Kat Reich, Kristefer Stojanovski

**Affiliations:** 1 School of Public Health, San Diego State University, San Diego, CA USA; 2 Division of Infections Disease and Global Public Health La Jolla, Herbert Wertheim School of Public Health and Human Longevity Science, University of California San Diego, 9500, Gilman Drive, La Jolla, 92093 USA; 3 Department of Epidemiology, Celia Scott Weatherhead School of Public Health and Tropical Medicine, Tulane University, New Orleans, LA USA; 4 Celia Scott Weatherhead School of Public Health and Tropical Medicine, Tulane University, New Orleans, LA USA; 5 Rudolph Matas Library of Health Sciences, Tulane University, New Orleans, LA USA; 6 Trac B Exchange, Las Vegas, NV USA; 7 Department of Social, Behavioral, and Population Sciences, Celia Scott Weatherhead School of Public Health and Tropical Medicine, Tulane University, New Orleans, LA USA

**Correction to: Harm Reduct J (2026) 23:56** 10.1186/s12954-025-01376-9

In this article [1], the authors had identified that Fig. 2 was inadvertently included and now it has been removed.

The original article has been corrected.

## References

[1] Nacht CL, Skaathun B, Ogarrio K, et al. Barriers and facilitators to initiating and adhering to harm reduction services among people who inject drugs in the United States: a systematic review. Harm Reduct J. 2026;23:56. 41680818 10.1186/s12954-025-01376-9 PMC13005311

