# Supplementary material for: Barriers and facilitators to initiating and adhering to harm reduction services among people who inject drugs in the United States: a systematic review
Source: Harm Reduct J. 2026 Feb 12;23:56. doi: 10.1186/s12954-025-01376-9 (PMC13005311; doi:10.1186/s12954-025-01376-9)
Supplement: Supplementary file 1 — Additional file1. [file 12954_2025_1376_MOESM1_ESM.docx]

**Supplementary Table 1.** Search terms and results

| **Database** | **Date of search** | **Search terms** | **Results** |
| --- | --- | --- | --- |
| PubMed | June 3, 2024 | "needle exchange"[All Fields] OR "needle exchanges"[All Fields] OR "syringe exchange"[All Fields] OR "syringe exchanges"[All Fields] OR "drug testing"[All Fields] OR "drug screening"[All Fields] OR "Direct-To-Consumer Screening and Testing"[MeSH Terms] OR "Substance Abuse Detection"[MeSH Terms] OR "Needle-Exchange Programs"[MeSH Terms] OR "harm reduction"[All Fields] OR "drug checking"[All Fields] OR "drug checking service"[All Fields] OR "opioid agonist"[All Fields] OR "naloxone"[All Fields] OR "drug overdose/prevention and control"[MeSH Terms] OR "point of care"[All Fields] OR "drug detection"[All Fields] OR "Harm Reduction"[Mesh] AND "Health Disparate Minority and Vulnerable Populations"[Mesh] | 751 |
| Scopus | June 6, 2024 | ( "needle exchange" OR "needle exchanges" ) OR ( "syringe exchange" OR "syringe exchanges" ) OR ( "drug screening" OR "drug testing" OR "direct-to-consumer screening and testing" ) OR ( "Substance Abuse Detection" OR "Needle-Exchange Programs" ) OR ( "harm reduction" OR "drug checking" OR "drug checking service" ) OR ( "opioid agonist" OR "naloxone" OR "drug overdose/prevention and control" ) OR ( "point of care" OR "drug detection" ) AND ( "Health Disparate Minority and Vulnerable Populations" OR "LGBTQ+ population" OR "Minority group" ) | 887 |
| CINAHL | June 3, 2024 | ("LGBTQ+ Persons") OR ("Minority Groups") OR ("Sexual and Gender Minorities") OR (MH "Low Socioeconomic Status") OR ("Special Populations") AND ("Harm Reduction") OR ("Needle Exchange Programs") OR ("Substance Abuse Detection") OR ("drug checking") OR ("Naloxone") | 158 |
| Web of Science | June 6, 2024 | "needle ( "needle exchange" OR "needle exchanges" ) OR ( "syringe exchange" OR "syringe exchanges" ) OR ( "drug screening" OR "drug testing" OR "direct-to-consumer screening and testing" ) OR ( "Substance Abuse Detection" OR "Needle-Exchange Programs" ) OR ( "harm reduction" OR "drug checking" OR "drug checking service" ) OR ( "opioid agonist" OR "naloxone" OR "drug overdose/prevention and control" ) OR ( "point of care" OR "drug detection" ) AND ( "Health Disparate Minority and Vulnerable Populations" OR "LGBTQ+ population" OR "Minority group" )exchange" OR "needle exchanges" (Abstract) | 78 |
| Total |  |  | 1788 |
